# TSH index and 24-hour mean heart rate in euthyroid adults: opposing FT3 and heart rate variability components

**DOI:** 10.3389/fendo.2026.1863377

**Published:** 2026-08-05

**Authors:** Yingyue Zhang, Guojie Ye, Wenchang Zhang, Changjian He, Peng Wang, Chunhua Ding

**Affiliations:** Department of Cardiology, Aerospace Center Hospital, Beijing, China

**Keywords:** autonomic regulation, euthyroid, heart rate variability, mean heart rate, mediation analysis, thyroid hormone sensitivity, TSH index

## Abstract

**Background:**

Central thyroid homeostasis may contribute to variation in heart rate even within the euthyroid range, but the relative contributions of endocrine and autonomic correlates remain uncertain.

**Objective:**

To examine the association between the TSH index (TSHI) and 24-hour mean heart rate and to explore whether this association could be statistically decomposed into components involving free triiodothyronine (FT3) and heart rate variability.

**Methods:**

This cross-sectional observational study was conducted as a secondary analysis of a previously described cohort. The cohort comprised 550 euthyroid adults who underwent 24-hour ambulatory electrocardiography and contemporaneous thyroid function testing. The present analysis focused specifically on the association between TSHI and 24-hour mean heart rate and its statistical decomposition involving FT3 and ln(SDNN). Multivariable linear regression and parallel mediation analysis, interpreted as statistical decomposition rather than causal mediation, were used after adjustment for demographic factors, body mass index, blood pressure, lifestyle factors, and major cardiometabolic comorbidities.

**Results:**

Higher TSHI was associated with lower mean heart rate (β −3.488 bpm per 1-unit increase; 95% CI −4.633 to −2.343; P < 0.001). Parallel mediation suggested opposite-direction indirect components: a positive association through FT3 (indirect estimate +0.251 bpm; 95% CI 0.052 to 0.520) and a negative indirect association through ln(SDNN) (indirect estimate −0.607 bpm; 95% CI −1.219 to −0.039). The net indirect association was not significant (−0.356 bpm; 95% CI −0.998 to 0.270), whereas the residual direct association remained predominant (c′ −3.132 bpm; 95% CI −4.076 to −2.138).

**Conclusion:**

In euthyroid adults, the association between TSHI and 24-hour mean heart rate appears to comprise FT3 and heart rate variability components in opposite directions, while the residual direct association remains predominant. These findings should be interpreted as reflecting cross-sectional statistical decomposition, rather than providing evidence for causal mediation.

## Background

Heart rate is a clinically informative cardiovascular measure that is closely linked to adverse cardiovascular outcomes (1, 2). Heart rate reflects intrinsic sinoatrial node automaticity as well as superimposed neurohumoral influences (3–6). Twenty-four-hour mean heart rate captures day-night physiology and is less sensitive to situational influences than a single clinic measurement. Prospective data further suggest that higher ambulatory mean heart rate predicts mortality independently of resting clinic heart rate and conventional risk factors (7).

The hypothalamic-pituitary-thyroid axis is a plausible contributor to inter-individual variation in heart rate through direct electrophysiologic effects and interactions with autonomic regulation (3, 4). Recent work has extended this focus from isolated thyroid hormone concentrations to composite indices of central thyroid homeostasis and feedback regulation (8–10). One such metric is the TSH index (TSHI), an FT4-adjusted TSH-based index that has been used as a proxy of pituitary feedback set-point regulation (8). Focusing on TSHI therefore allowed us to further examine central thyroid feedback regulation in relation to heart rate, building on our previous finding of an association between TSHI and heart rate. In this context, even when thyroid-related indices are associated with heart rate, it remains unclear whether these associations are fully explained by circulating active hormone availability (11, 12).

Thyroid signaling has established cardiovascular effects, including chronotropic and electrophysiologic changes that may influence heart rate (4, 6, 13–15). Beyond direct effects on the sinus node and myocardium, thyroid hormone signaling may also interact with autonomic cardiovascular regulation by modulating sympathetic responsiveness and neurohumoral control of heart rate. At the same time, heart rate may also reflect longer-term regulatory influences that are not fully captured by circulating active hormone levels alone. A related challenge is that many thyroid-autonomic studies rely on short HRV recordings, often around 5 minutes, whereas long-term indices such as standard deviation of normal-to-normal (SDNN) require extended monitoring and capture a different level of autonomic integration (16–19). HRV standards likewise emphasize that SDNN is a global time-domain measure best interpreted from extended recordings and conceptually distinct from short-term vagal indices (19). SDNN was therefore selected because it is a standard long-term time-domain HRV parameter available from 24-hour Holter monitoring and reflects overall variability across the same recording period used to calculate mean heart rate (3, 4, 6). However, HRV indices are intrinsically coupled to mean heart rate through both physiological and mathematical dependence, so their role as intermediate measures requires cautious interpretation (20). Together, these considerations suggest that the association between TSHI and mean heart rate may arise not only from circulating active thyroid hormone availability, but also from longer-term regulatory influences. This highlights a specific gap in understanding whether the association between TSHI and mean heart rate can be separated into FT3-related and SDNN-related components within a parallel statistical decomposition framework.

In this study, we examined this question in euthyroid adults using a parallel mediation framework as an exploratory statistical decomposition. We prespecified FT3 and ln(SDNN) as variables in the parallel mediation model. FT3 was intended to reflect circulating active hormone availability, whereas SDNN derived from 24-hour Holter monitoring was used as a long-term HRV measure. We aimed to assess whether FT3 and SDNN contributed to the association between TSHI and mean heart rate.

## Materials and methods

This cross-sectional secondary analysis used a previously described euthyroid cohort of 550 adults who underwent routine health evaluation with at least 20 hours of ambulatory electrocardiography (Holter monitoring) and contemporaneous thyroid function testing at Aerospace Center Hospital between January 2022 and September 2023. Eligibility criteria, definition of euthyroidism, and exclusion criteria were consistent with the prior report and are described below (10). Eligible participants were adults aged ≥18 years who underwent routine physical examination, ambulatory electrocardiography with a recording duration of more than 20 hours, and contemporaneous thyroid function testing. Euthyroidism was defined as FT3 2.76–6.45 pmol/L, FT4 11.20–23.81 pmol/L, TT3 0.89–2.49 nmol/L, TT4 64.35–186.62 nmol/L, and TSH 0.35–5.10 mIU/L, without a known history of thyroid disease or treatment for thyroid disease.

Participants were excluded if they had non-sinus rhythm on ambulatory electrocardiography, severe hepatic or renal dysfunction, history of cancer, acute cardiac insufficiency, acute myocardial infarction, anemia, acute infection, abnormal thyroid function, subclinical thyroid dysfunction, history of thyroid disease, antithyroid therapy, hormone replacement therapy, or incomplete data. Participants using medications known to influence heart rate were also excluded, including class I, II, III, and IV antiarrhythmic drugs, ivabradine, and cardiac glycosides, with representative examples including propafenone, metoprolol, diltiazem, amiodarone, ivabradine, and digoxin. Finally, 550 participants were included in the present analysis.

The present analysis examined the association between TSHI and mean heart rate and further assessed whether this association could be statistically decomposed into FT3-related and HRV-related components. The study was approved by the Ethics Committee of Aerospace Center Hospital (2023 No. 132). The study was reported in accordance with the Strengthening the Reporting of Observational Studies in Epidemiology (STROBE) guidelines.

### Exposure, outcome, intermediate variables, and covariates

Central thyroid homeostasis was indexed by TSHI, calculated as ln[TSH (mIU/L)] + 0.1345 × FT4 (pmol/L). This index was originally proposed as an FT4-adjusted TSH-based metric reflecting the pituitary thyrotroph set point under negative feedback. Higher values indicate higher FT4-adjusted TSH and are generally interpreted as reflecting lower central feedback sensitivity.

The primary outcome was Holter-derived mean heart rate (bpm), calculated from approximately 24-hour ambulatory electrocardiography recordings with at least 20 hours of valid data. Two prespecified intermediate variables were FT3 (pmol/L) and SDNN (ms), a time-domain heart rate variability metric defined as the standard deviation of normal-to-normal intervals across the recording and commonly used as a measure of overall HRV in long-term ECG monitoring.

Because SDNN was right-skewed, it was natural log-transformed and entered as ln(SDNN) in the statistical decomposition model.

Fully adjusted models included age, sex, body mass index (BMI), systolic blood pressure (SBP), diastolic blood pressure (DBP), smoking, alcohol drinking, coronary heart disease (CHD), hypertension (HTN), and type 2 diabetes mellitus (T2DM), as recorded in the source dataset.

### Statistical analysis

Continuous variables are summarized as mean (SD), and categorical variables as n (%). Participants with incomplete data required for the present analysis were excluded during cohort construction, consistent with the prior report (10).

The association between TSHI and mean heart rate was evaluated using multivariable linear regression with three models: Model 1 (unadjusted), Model 2 (adjusted for age and sex), and Model 3 (fully adjusted for age, sex, BMI, SBP, DBP, smoking, alcohol drinking, CHD, HTN, and T2DM). Regression coefficients are reported as β (bpm per 1-unit higher TSHI) with 95% confidence intervals (CIs). Because heteroskedasticity may affect standard errors in observational regression models, inference was based on HC3 heteroskedasticity-consistent standard errors to improve robustness. Model diagnostics were reviewed to assess linearity, residual distribution, influential observations, and multicollinearity using residual plots, Cook’s distance, and variance inflation factors.

To facilitate comparison of effect sizes, standardized estimates per 1-SD higher TSHI were additionally obtained by z-standardizing TSHI and refitting the same three models.

Statistical decomposition was assessed using parallel mediation analysis implemented as a path-analytic structural equation model. FT3 and ln(SDNN) were entered simultaneously as intermediate variables. The mediator models specified FT3 and ln(SDNN) as dependent variables, each regressed on TSHI and the covariates included in Model 3. The outcome model specified mean heart rate as the dependent variable, with TSHI, FT3, ln(SDNN), and the same covariates entered simultaneously. Indirect associations were quantified as a1×b1 for FT3 and a2×b2 for ln(SDNN), with total indirect and total associations derived from these components. Because the sampling distribution of indirect association estimates is often asymmetric, uncertainty was quantified using 5,000 nonparametric bootstrap samples and percentile 95% confidence intervals, a resampling number selected to provide stable interval estimation while maintaining computational feasibility.

Prespecified subgroup analyses examined the fully adjusted association between TSHI and mean heart rate across strata defined by sex, T2DM, HTN, and CHD. In each subgroup model, the stratifying variable was omitted, and all remaining prespecified covariates were retained. Potential effect modification by sex and T2DM was additionally evaluated using multiplicative interaction terms (TSHI×sex and TSHI×T2DM) in fully adjusted models, with results reported in **Supplementary Table 1**.

All analyses were performed using R version 4.4.0. Parallel mediation analysis was implemented using the lavaan package version 0.6.17 in R. All statistical tests were two-sided, and P < 0.05 was considered statistically significant.

## Results

### Study population

A total of 550 euthyroid adults were included. Baseline characteristics are summarized in **Table 1**. The mean age was 62.03 years, and 55.8% were female. Mean heart rate was 67.85 bpm, and mean SDNN was 121.94 ms. Thyroid measures, including TSH, FT3, FT4, and TSHI, were available for the prespecified analyses.

**Table 1:** Baseline characteristics of the study population (N = 550).

Variable	Overall
**Age (years)**	62.03 (13.94)
Sex	
Male	243 (44.2%)
Female	307 (55.8%)
**BMI (kg/m²)**	25.56 (3.77)
**SBP (mmHg)**	135.79 (19.39)
**DBP (mmHg)**	80.15 (12.69)
Smoking	
No	443 (80.5%)
Yes	107 (19.5%)
Alcohol drinking	
No	438 (79.6%)
Yes	112 (20.4%)
Coronary heart disease	
No	336 (61.1%)
Yes	214 (38.9%)
Hypertension	
No	282 (51.3%)
Yes	268 (48.7%)
Type 2 diabetes mellitus	
No	486 (88.4%)
Yes	64 (11.6%)
**TSH (mIU/L)**	2.16 (1.05)
**FT4 (pmol/L)**	16.62 (2.55)
**FT3 (pmol/L)**	4.14 (0.56)
**TSHI**	2.88 (0.56)
**Mean heart rate (bpm)**	67.85 (8.47)
**Minimum heart rate (bpm)**	49.91 (7.13)
**Maximum heart rate (bpm)**	107.74 (15.52)
**SDNN (ms)**	121.94 (60.98)

Continuous variables are presented as mean (SD) and categorical variables as n (%). Abbreviations: BMI, body mass index; SBP, systolic blood pressure; DBP, diastolic blood pressure; TSH, thyroid-stimulating hormone; FT4, free thyroxine; FT3, free triiodothyronine; TSHI, thyroid-stimulating hormone index; SDNN, standard deviation of all normal-to-normal intervals.

Association between TSHI and mean heart rate.

Higher TSHI was consistently associated with lower 24-hour mean heart rate across all three regression models (**Table 2**). In the fully adjusted model, each 1-unit higher TSHI was associated with a 3.488 bpm lower mean heart rate (β −3.488, 95% CI −4.633 to −2.343; P < 0.001). The association was similar when TSHI was analyzed per 1-SD increment.

**Table 2:** Multivariable linear regression of mean heart rate on TSHI.

Model	β per 1-unit TSHI (95% CI)	P value (1-unit)	β per 1-SD TSHI (95% CI)	P value (1-SD)	Adjusted R²
Model 1	-3.600 (-4.791, -2.410)	<0.001	-2.011 (-2.676, -1.346)	<0.001	0.055
Model 2	-3.608 (-4.752, -2.463)	<0.001	-2.015 (-2.655, -1.376)	<0.001	0.133
Model 3	-3.488 (-4.633, -2.343)	<0.001	-1.948 (-2.588, -1.309)	<0.001	0.156

β estimates and 95% CIs were derived from linear regression using HC3 heteroskedasticity-consistent (robust) standard errors. Per 1-SD TSHI estimates were obtained by z-standardizing TSHI and refitting each model and are provided to facilitate comparison of effect sizes. Adjusted R² refers to the corresponding regression model.

Model 1 unadjusted;

Model 2 was adjusted for age and sex;

Model 3 was adjusted for age, sex, BMI, SBP, DBP, smoking, alcohol drinking, CHD, HTN, and T2DM.

On a standardized scale, each 1-SD higher TSHI was associated with a 1.948-bpm lower mean heart rate in the fully adjusted model (95% CI, −2.588 to −1.309; P < 0.001) (**Table 2**). Regression diagnostics did not indicate problematic multicollinearity or highly influential observations; all variance inflation factors in the fully adjusted model were below 2, and the maximum Cook’s distance was 0.024.

### Parallel mediation decomposition

**Figure 1:**
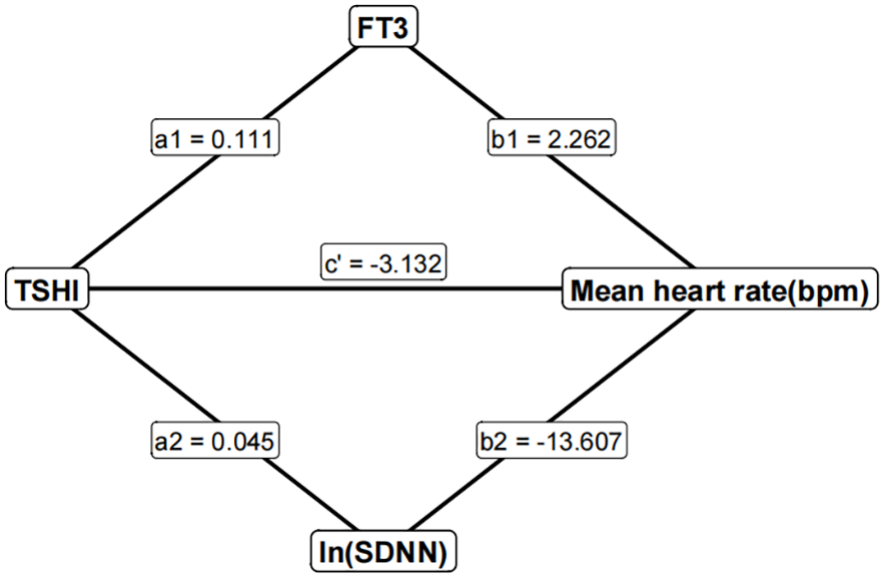
Parallel mediation model for the association between TSHI and mean heart rate. Path diagram of the parallel mediation model with FT3 and ln(SDNN) entered simultaneously as mediators of the association between TSHI and mean heart rate. Path coefficients are shown for the fully adjusted model; covariates included in all equations are omitted for clarity. The c path represents the total association between TSHI and mean heart rate, whereas c′ represents the residual direct association after simultaneous inclusion of FT3 and ln(SDNN). Indirect components were estimated as a1×b1 for the FT3-related path and a2×b2 for the SDNN-related path.

The TSHI-mean heart rate association was next decomposed into parallel indirect components involving FT3 and ln(SDNN), modeled simultaneously (**Figure 1**; **Table 3**). In the adjusted mediation model, TSHI was positively associated with both FT3 (a1 = 0.111, 95% CI, 0.029 to 0.194) and ln(SDNN) (a2 = 0.045, 95% CI, 0.003 to 0.087). FT3 was positively associated with mean heart rate (b1 = 2.262, 95% CI, 0.984 to 3.521), whereas ln(SDNN) was inversely associated with mean heart rate (b2 = −13.607, 95% CI, −16.557 to −10.999) (**Table 3**).

**Table 3:** Parallel mediation model for the association between TSHI and mean heart rate.

Effect	Estimate (95% CI)	P value	Std. β
a1: TSHI → FT3	0.111 (0.029, 0.194)	0.00823	0.111
a2: TSHI →ln(SDNN)	0.045 (0.003, 0.087)	0.03608	0.082
c′: Residual direct association (TSHI → mean heart rate)	-3.132 (-4.076, -2.138)	< 0.001	−0.206
b1: FT3 → mean heart rate	2.262 (0.984, 3.521)	< 0.001	0.149
b2: ln(SDNN) → mean heart rate	-13.607 (-16.557, -10.999)	< 0.001	−0.490
Indirect association via FT3 (a1×b1)	0.251 (0.052, 0.520)	0.03727	0.017
Indirect association via ln(SDNN) (a2×b2)	-0.607 (-1.219, -0.039)	0.04040	−0.040
Total indirect association	-0.356 (-0.998, 0.270)	0.26137	−0.023
Total association	-3.488 (-4.602, -2.316)	< 0.001	−0.230

Covariates (age, sex, BMI, SBP, DBP, smoking, alcohol drinking, CHD, HTN, and T2DM) were included in all intermediate variables and outcome equations. SDNN was natural log-transformed and entered as ln(SDNN). Percentile bootstrap 95% confidence intervals for indirect associations were obtained from 5,000 bootstrap samples. The confidence interval for the total association was estimated within the mediation/SEM framework and may therefore differ slightly from the HC3 robust confidence interval in **Table 2**. Abbreviations: SDNN, standard deviation of normal-to-normal intervals.

**Figure 2:**
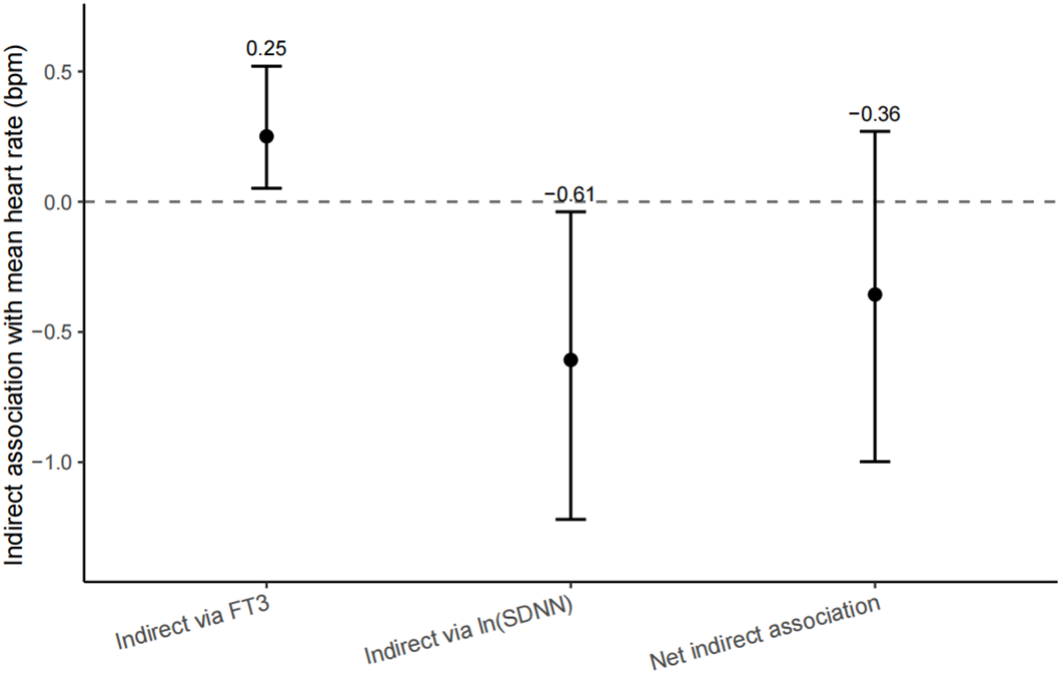
Decomposition of indirect associations through FT3 and the ln(SDNN). Decomposition of the indirect associations between TSHI and mean heart rate through FT3 and the ln(SDNN), together with the net indirect association. Points indicate indirect association estimates (bpm), and lines indicate 95% bootstrap confidence intervals from 5,000 resamples. FT3-related and SDNN-related indirect components were estimated simultaneously in the adjusted parallel model.

The residual direct association remained negative (c′ = −3.132 bpm, 95% CI −4.076 to −2.138; P < 0.001) (**Table 3**). Indirect estimates showed opposing directions: the indirect association via FT3 was positive (+0.251 bpm, 95% CI 0.052 to 0.520), whereas the indirect association via ln(SDNN) was negative (−0.607 bpm, 95% CI −1.219 to −0.039) (**Figure 2**; **Table 3**). These opposing-direction components partially offset each other, yielding a non-significant net indirect estimate of −0.356 bpm (95% CI −0.998 to 0.270) despite a significant total association (**Table 3**).

### Prespecified subgroup analyses

**Figure 3:**
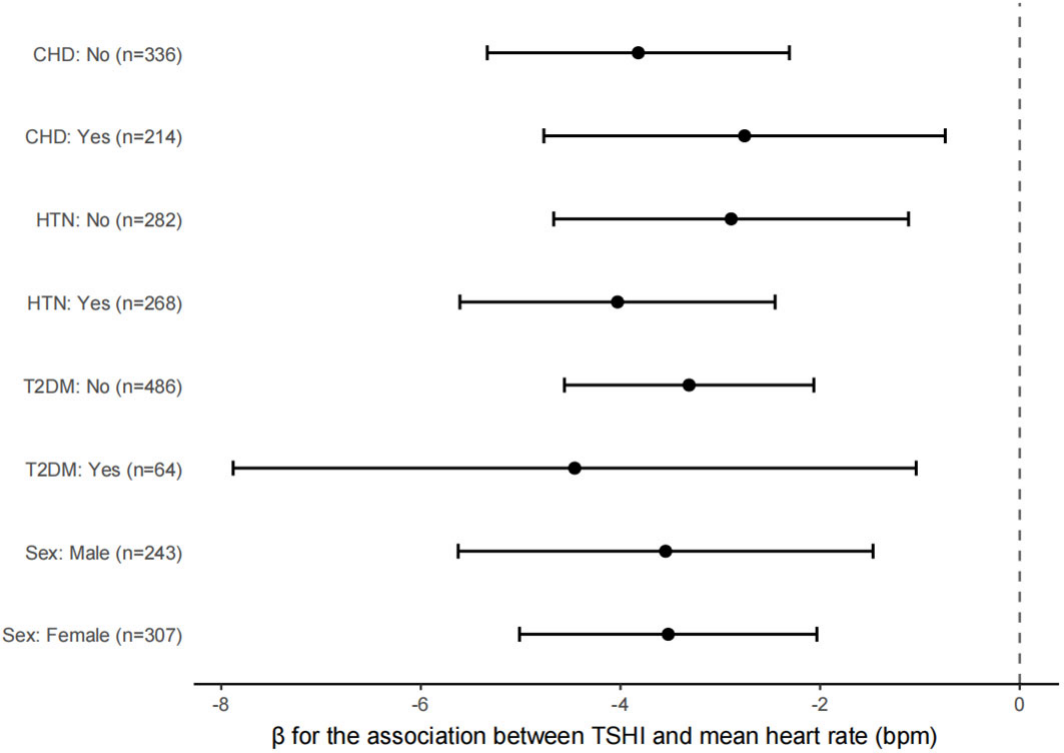
Subgroup analyses of the association between TSHI and mean heart rate. Forest plot of subgroup-specific associations between TSHI and mean heart rate from fully adjusted linear regression models stratified by sex, T2DM, HTN, and CHD. Points indicate β estimates, and horizontal lines indicate 95% confidence intervals.

The fully adjusted TSHI–mean heart rate association was examined across prespecified clinical subgroups stratified by sex, T2DM, HTN, and CHD (**Figure 3**). Effect estimates were directionally consistent across strata. Formal interaction testing showed no evidence of effect modification by sex (P for interaction = 0.933) or T2DM (P for interaction = 0.528) (**Supplementary Table 1**).

## Discussion

Consistent with our prior report, higher TSHI was associated with a lower 24-hour mean heart rate in this cohort. The main additional finding here was that the FT3-related and HRV-related components pointed in opposite directions. The indirect estimate through FT3 was positive (+0.251 bpm; 95% CI 0.052 to 0.520), whereas the corresponding estimate through ln(SDNN) was negative (−0.607 bpm; 95% CI −1.219 to −0.039). These opposing components partially offset each other (net indirect −0.356 bpm; 95% CI −0.998 to 0.270), while the residual direct association remained negative and larger in magnitude (c′ −3.132 bpm; 95% CI −4.076 to −2.138; P < 0.001).

Although the overall inverse association is directionally consistent with prior reports (9, 10, 21), the principal contribution of the present study lies in its statistical decomposition into FT3-related and HRV-related components with opposite directions. These indirect components should be interpreted as statistical decomposition of cross-sectional associations rather than evidence of biological mediation.

The FT3-related component is physiologically plausible. Thyroid hormones exert direct genomic and non-genomic chronotropic effects and modulate β-adrenergic responsiveness, so higher effective thyroid signaling is typically associated with higher heart rate (3, 4, 6, 13). In this context, triiodothyronine is generally regarded as the more biologically active mediator of thyroid hormone effects on heart rate (22, 23). In euthyroid populations, circulating FT3 within the reference range has been reported to correlate positively with heart rate (12, 24), which is consistent with the positive FT3-HR association observed in our parallel mediation model. This suggests that the FT3-related component is consistent with circulating active thyroid hormone availability.

The HRV-related component is also directionally plausible. Higher long-term HRV is commonly associated with lower average heart rate, consistent with the negative SDNN-related component observed in our model (25). Consistent with this, the use of SDNN as a long-term HRV metric is grounded in established HRV standards (19). Interpretation of the SDNN-related component should nevertheless acknowledge the dependence of HRV measures on mean heart rate. Because SDNN and mean heart rate are both derived from normal-to-normal intervals, this component may partly reflect mathematical and physiological dependence between HRV measures and heart rate, rather than autonomic regulation alone (20).

Taken together, the findings support a working model in which the association between TSHI and mean heart rate comprises at least two statistically distinguishable components. One is aligned with FT3, and the other with ln(SDNN). The coexistence of these opposite-direction indirect estimates provides quantitative support for a possible endocrine-autonomic counterbalance pattern. The remaining direct association suggests that additional factors may also contribute to the observed relationship (5, 26, 27).

Variation in heart rate within the euthyroid range may reflect differences in thyroid homeostatic regulation rather than random physiological variation alone (9, 10). Recent studies linking thyroid hormone sensitivity or euthyroid thyroid hormone variation with cardiovascular events, diastolic dysfunction, cardiovascular risk profiles, valvular heart disease, and atrial fibrillation support this interpretation (21, 22, 28–30). The coexistence of opposite-direction FT3-related and HRV-related components highlights the need to consider multi-system regulation when interpreting heart rate variation, particularly in clinical contexts such as sinus bradycardia where conventional thyroid function tests are often unremarkable (28, 29).

Several limitations should be considered. The cross-sectional design precludes establishing temporal ordering among TSHI, FT3, SDNN, and mean heart rate, so mediation should be interpreted as statistical decomposition rather than causal mediation (26, 31). SDNN is a global long-term HRV measure and does not substitute for more specific parasympathetic markers. Although participants using major heart-rate-modifying medications were excluded, including class I–IV antiarrhythmic drugs, information on other medications and some factors that may influence heart rate, such as physical activity and sleep status, was not fully available. Therefore, residual confounding cannot be completely excluded. Because SDNN and mean heart rate were both derived from Holter NN-interval information, the SDNN-related component may partly reflect mathematical or physiological coupling rather than a distinct autonomic mechanism. Heart-rate-corrected or heart-rate-independent HRV measures were not available, and this component should therefore be interpreted cautiously. In addition, TSHI is only one of several indices used to characterize thyroid hormone sensitivity or feedback regulation, rather than a direct measure of tissue-level thyroid hormone sensitivity. Alternative indices such as TT4RI, TFQI, and PTFQI may reflect partly different aspects of thyroid homeostasis (8–10). Therefore, TSHI should be considered a candidate marker of central thyroid feedback-related heart rate variation rather than a validated clinical biomarker. Selection bias is possible because participants were derived from a single-center health evaluation cohort with available Holter monitoring and thyroid function testing, which may limit generalizability. Measurement variability is also possible because thyroid function was assessed at a single time point.

Further studies are needed to test and refine this interpretation. Multicenter validation would strengthen external validity, and longitudinal designs could clarify temporal ordering. Mechanistic studies using HRV measures corrected for heart rate and complementary autonomic measures may further disentangle physiological regulation from mathematical heart rate-HRV coupling.

In euthyroid adults, parallel mediation suggested two indirect components with opposite directions in the association between TSHI and 24-hour mean heart rate. The FT3-related component was positive, whereas the ln(SDNN)-related component was negative. The residual direct association remained the predominant component after accounting for these intermediate measures. Taken together, these findings are consistent with an endocrine-autonomic counterbalance framework for interpreting heart rate variation within the euthyroid range. They remain compatible with a non-causal interpretation given the cross-sectional design.

## Data Availability

The dataset is subject to restrictions due to patient privacy, institutional data protection policies, and ethics requirements. Therefore, it is not publicly available. Requests to access the datasets should be directed to Chunhua Ding, dingmd@gmail.com.

## Supplementary material

Supplementary Table 1 Interaction analyses for the association between TSHI and mean heart rate. P for interaction was derived from the multiplicative interaction term in the fully adjusted linear regression model, with HC3 robust standard errors. Sex was coded as female versus male.
