# Supplementary material for: TSH index and 24-hour mean heart rate in euthyroid adults: opposing FT3 and heart rate variability components
Source: Front Endocrinol (Lausanne). 2026 Aug 5;17:1863377. doi: 10.3389/fendo.2026.1863377 (PMC13485541; doi:10.3389/fendo.2026.1863377)
Supplement: Supplementary file 1 [file Table1.docx]

**Supplementary Table 1.** Interaction analyses for the association between TSHI and mean heart rate

| Interaction | Interaction β (95% CI) | P for interaction |
| --- | --- | --- |
| TSHI × sex | 0.104 (-2.338, 2.546) | 0.933 |
| TSHI × T2DM | -1.123 (-4.618, 2.372) | 0.528 |

*P for interaction was derived from the multiplicative interaction term in the fully adjusted linear regression model, with HC3 robust standard errors.* *Sex was coded as female versus male.*
